# Immunosuppressive, anti-inflammatory and anti-cancer properties of triptolide: A mini review

**Published:** 2016

**Authors:** Samira Ziaei, Reginald Halaby

**Affiliations:** Montclair State University, Department of Biology, 1 Normal Avenue, Montclair NJ 07043, USA

**Keywords:** *Triptolide*, *Immunosuppression*, *Anti-inflammation*, *Autoimmune disorders*, *Cancer*

## Abstract

**Objective:**

Triptolide, the active component of *Tripterygium wilfordii *Hook F has been used to treat autoimmune and inflammatory conditions for over two hundred years in traditional Chinese medicine. However, the processes through which triptolide exerts immunosuppression and anti-inflammation are not understood well. In this review, we discuss the autoimmune disorders and inflammatory conditions that are currently treated with triptolide. Triptolide also possesses anti-tumorigenic effects. We discuss the toxicity of various triptolide derivatives and offer suggestions to improve its safety. This study also examines the clinical trials that have investigated the efficacy of triptolide. Our aim is to examine the mechanisms that are responsible for the immunosuppressive, anti-inflammatory, and anti-cancer effects of triptolide.

**Materials and Methods:**

The present review provides a comprehensive summary of the literature with respect to the immunosuppressive, anti-inflammatory, and anti-cancer properties of triptolide.

**Results:**

Triptolide possesses immunosuppressive, anti-inflammatory, and anti-cancer effects.

**Conclusion:**

Triptolide can be used alone or in combination with existing therapeutic modalities as novel treatments for autoimmune disorders, cancers, and for immunosuppression.

## Introduction

Although inflammation is important in preventing disease, there are numerous autoimmune disorders that involve deleterious inflammatory responses, including rheumatoid arthritis, systemic lupus erythematosus, multiple sclerosis, psoriasis, and Type 1 diabetes. Autoimmune disorders are varied and, as a result they often require disease-specific treatments. Many of these treatments, however, have untoward side effects including immunodeficiency, hypertension, hyperglycemia, bleeding, anemia, and life-threatening allergic reactions. Clearly, the development of safer and more effective therapies for autoimmune diseases is warranted. In this report, we investigate the use of triptolide, an extract of the Chinese herb *Tripterygium wilfordii* Hook F. (TWHF) and the bioactive component of TWHF as a natural anti-autoimmune agent.

 Triptolide is a woody vine which is widely distributed in Eastern and Southern China. In China, triptolide is frequently used to treat autoimmune and/or inflammatory diseases due to its favorable cost–benefit ratio. Commercial preparations of triptolide have been commonly used for the treatment of inflammatory and autoimmune diseases such as rheumatoid arthritis, systemic lupus erythematosus, nephritis and psoriasis (Tao and Lipsky, 2000 ▶; Qiu and Kao, 2003 ▶; Zheng et al., 2008 ▶). Triptolide has been demonstrated to exert novel chondroprotective and anti-inflammatory effects on rheumatoid arthritis (Lin et al., 2007 ▶).

Although triptolide has been used in traditional Chinese medicine for over two centuries, until recently, few clinical trials have been conducted in the United States to determine its safety and efficacy. Two recent small clinical studies have demonstrated tiptolide’s effectiveness against rheumatoid arthritis (Tao et al., 2001 ▶; Tao et al., 2002 ▶). A larger study confirmed the therapeutic effects of triptolide in the aforementioned studies (Goldbach-Mansky et al., 2009 ▶). Goldbach-Mansky et al. (2009) ▶ demonstrated that triptolide was more effective than sulfasalazine for treatment ofthe symptoms associated with rheumatoid arthritis. 

Triptolide is among the most powerful and broadly active anti-inflammatory/immunomodulating natural products ever discovered (Graziose et al., 2010 ▶). Triptolide acts at nanomolar concentrations and inhibits the production of various cellular targets including inflammatory cytokines (Wang et al., 2014 ▶), cyclooxygenase (Peng et al., 2014 ▶), inducible nitric oxide synthase (Kim et al., 2004 ▶; Wang et al., 2004a ▶; Tong et al., 2007 ▶), and metalloproteinases (Liacini et al., 2005 ▶; Lin et al., 2007 ▶) and transcription factors (Wang et al., 2004a ▶; Wei and Huang, 2014 ▶).

 It has been suggested that triptolide is a very effective alternative to conventional drug-based treatments for autoimmune disorders, possibly with fewer side effects. Likewise, we describe how scientists are modifying the molecular structure of triptolide with the goal of producing safer analogues while retaining the same or improved immunosuppressive and anti-inflammatory efficacy. This report will examine the effects of triptolide as a treatment modality for various autoimmune diseases and propose putative molecular pathways to account for its diverse anti-inflammatory actions. Lastly, we will provide data from our laboratory that shows triptolide induces lysosomal-mediated apoptosis (Owa et al., 2013 ▶). Deregulated apoptosis has been implicated in the pathogenesis of many autoimmune diseases. Despite the vast research describing the anti-inflammatory and immunosuppressive effects of triptolide, the molecular mechanisms that regulate these actions are poorly understood. This study will shed valuable insights that will contribute to our understanding of triptolide’s mode of action.

**Triptolide derivatives**

The poor water solubility of triptolide limits its clinical effectiveness. For example, intravenous injections of triptolide require formulation of the compound with a solubilizing reagent such as Cremophor EL (Wong et al., 2012 ▶). However, the use of Cremophor EL in some patients has been associated with important clinical complications such as severe anaphylactoid hypersensitivity reactions, hyperlipidaemia, abnormal lipoprotein patterns, aggregation of erythrocytes and peripheral neuropathy (Gelderblom et al., 2001 ▶). In light of this therapeutic constraint, several triptolide derivatives with improved water solubility have been developed, namely PG490-88, LLDT-8, and F60008 (Wong et al., 2012 ▶). PG490-88 has been shown to have anti-inflammatory and anti-cancer effects that are comparable to those of triptolide (Pan et al., 2005 ▶). PG490-88 has been shown to prolong renal allograft survival in animal models (Wang et al., 2005 ▶) and prevent rejection in renal allografts (Pan et al., 2005 ▶). The immunosuppressive effect of PG490-88 is mediated by inhibition of alloreactive T cell expansion through interleukin-2 production (Chen et al., 2000 ▶). PG490-88 has also been shown to prevent rejection in other organ transplantation scenarios, including bleomycin-induced lung fibrosis (Krishna et al., 2001 ▶), bone marrow (Chen et al., 2002 ▶), treatment of obliterative airway disease following lung transplantation (Leonard et al., 2002 ▶). PG490-88 also possesses potent anti-cancer properties (Fidler et al., 2003 ▶). 

LLDT-8, also known as (5*R*)-5-hydroxytriptolide, is a novel triptolide derivative that mediates immunosuppression *in vitro* and *in vivo *(Zhou et al., 2005 ▶). Most importantly, compared to triptolide, LLDT-8 significantly reduced toxicity, with a 122-fold lower cytotoxicity *in vitro* and 10-fold lower acute toxicity *in vivo* (Zhou et al., 2005 ▶). It has been reported that the anti-arthritic effect of LLDT-8 is closely related to the blockade of IFN-gamma signaling (Zhou et al., 2006c ▶; Zhou et al., 2006b ▶). Therefore, LLDT-8 may be of therapeutic value in the treatment of rheumatoid arthritis. LLDT-8 has also been shown to prevent experimental autoimmune encephalomyelitis (EAE) by suppressing T cell proliferation and activation (Fu et al., 2006 ▶). These finding suggest that LLDT-8 may be a potential treatment for multiple sclerosis. The effects of LLDT-8 on concanavalin A-induced hepatitis were examined (Zhou et al., 2006a ▶). The protective effects of LLDT-8 involved elimination of activated T cells by increasing pro-apoptotic genes signal transducer and activator of transcription 1 (STAT1) and interferon regulatory factor-1 (IRF-1) expression in the spleen (Zhou et al., 2006a ▶).  LLDT-8 reduced production of IFN-gamma, IL-2, and tumor necrosis factor (TNF-alpha) peripheral blood mononuclear cells (Zhou et al., 2009 ▶).Clinical studies on LLDT-8 effects against rheumatoid arthritis are now being done in China (Liu et al., 2011 ▶).

MRx102 is another triptolide derivative (Carter et al., 2012 ▶; Fidler et al., 2014 ▶). This new formulation was shown to be more effective than triptolide when tested in acute myeloid leukemia patient cells (Fidler et al., 2014 ▶). Specifically, Fidler et al. (2014) ▶ demonstrated that this novel lipophilic triptolide analogue displayed a 20 to 60-fold reduction in toxicity compared to triptolide. The authors also showed that MRx102 administration resulted in a pharmacokinetic profile characterized by higher and more extended triptolide plasma levels *in vivo *compared to native triptolide injections. The mechanism of action of MRx102 appears to be mediated via inhibition of RNA synthesis and X-linked inhibitor of apoptosis protein (XIAP) protein, at least in leukemia cells (Carter et al., 2012 ▶).

**Anti-inflammatory properties of triptolide– mechanism of action**

Triptolide is a biologically active diterpenetriepoxide from the Chinese herb *T. wilfordii* Hook f. This natural product has been demonstrated to possess anti-inflammatory properties by numerous studies. Specifically, triptolide has been used in traditional Chinese medicine for a variety of inflammatory and autoimmune conditions such as rheumatoid arthritis, systemic lupus, psoriatic arthritis, and Behcet’s disease (Chen, 2001 ▶). In this section, we will review some of the main cellular pathways that are affected by triptolide to inhibit inflammation. Table 1 summarizes the molecular targets that mediate the anti-inflammatory activities of triptolide. 

The C-14 beta-hydroxyl and gamma butyrolactone moieties of the triptolide molecule were shown to be crucial for its anti-inflammatory properties and also for its cytotoxicity (Wong et al., 2008 ▶). Accessory cells, in particular professional APCs such as dendritic cells (DCs), are another target for the immunosuppressive activity of triptolide. Triptolide inhibited the differentiation, maturation, trafficking and function of immature DCs (Chen et al., 2005 ▶; Zhu et al., 2005 ▶). High concentrations (>20 ng/ml) of triptolide induced apoptosis in DCs through sequential p38MAP kinase phosphorylation and caspase-3 activation (Liu et al., 2004 ▶). DC-mediated chemo-attraction of neutrophils and T cells has also been shown to be inhibited by triptolide through decreased STAT3 phosphorylation and NF-kB activation (Liu et al., 2006 ▶). Triptolide prevented the differentiation of immature monocyte-derived DCs (MoDCs) by down-regulation of CD1a, CD40, CD80, CD86, and HLA-DR expression, and reducing the capacity of MoDCs to stimulate lymphocyte proliferation in the allogeneic mixed lymphocyte reaction (Han et al., 2012 ▶). 

Triptolide was shown to reduce IFN-gamma-induced CD80 and CD86 expressions and inhibit IL-12 and IL-23 expression in DCs and THP-1 cells, a human monocytic cell line (Chen et al., 2005 ▶; Liu et al., 2005 ▶; Liu et al., 2008 ▶). Recently, it was shown that triptolide inhibited Il-12/IL-23 expression in antigen presenting cells (APCs) via CCAAT/enhancer-binding protein alpha (Zhang and Ma, 2010 ▶), which provided mechanistic insights into the immunomodulatory capacity of triptolide. Triptolide has been shown to inhibit the secretion of IL-2, IL-4, IL-6, and IL-8 from monocytes (Chang et al., 1997 ▶). In addition, triptolide was shown to suppress pro-inflammatory responses by attenuating various intracellular signaling pathways including the toll like receptor (TLR) signaling. It was reported that treatment of mouse macrophages with triptolide suppressed inflammation by down-regulating TLR gene expression (Premkumar et al., 2010 ▶). Triptolide also blocks the production of two chemokines, IL-8 and MCP-1, in cultured human corneal fibroblasts stimulated with pro-inflammatory cytokines (Lu et al., 2005 ▶). 

The known mechanisms responsible for triptolide’s anti-inflammatory effects are as follows. In lung inﬂammation, triptolide (100–1,000 mg/kg for 6 hr) acted as a novel inhibitor of IL-8 and NF-κB expression by regulating substance P (IC50 of 23 nM and 14 nM, respectively) and inhibited the production of neutrophil chemoattractant KC in A549 cells (Hoyle et al., 2010 ▶). Moreover, triptolide (40–160 nM for 24 hr) inhibited NF-κB activation by increasing IκBα, an inhibitor of NF-κB, at mRNA and protein levels in multiple myeloma RPMI-8266 cells (Zeng et al., 2011 ▶). The cause of Crohn's disease (CD), which can cause dangerous enteritis in any part of the stomach, is still unclear. However, damage to the tight junction due to increased intestinal permeability is a symptom of CD (Ma et al., 2004 ▶). Triptolide (0.0035 mg/ml for 8 weeks) blunted the increase of TLR2/TLR4 in IL-10-deﬁceint C57/BL6 mice via TLR/NF-κB pathway, which is related to the pathogenesis of CD (Yu et al., 2011 ▶). Recent data demonstrated that triptolide (0.0035 mg/ml for 8 weeks) inhibited the expression of miR155, its downstream target, SHIP1, and inﬂammatory cytokines in IL-10 deﬁcient mice, reducing inﬂammation in CD (Wu et al., 2013a ▶). Triptolide modulates down-regulation of matrix metalloproteinases (MMPs). MMPs participate in tumorigenesis, tumor metastasis and inﬂammatory diseases such as rheumatoid arthritis. In human synovial ﬁbroblasts and mouse macrophages, Triptolide (28–140 nM for 24 hr) inhibited IL-1α- and LPS-induced phosphorylation of MMP1 and MMP3, respectively (Lin et al., 2001 ▶). Moreover, triptolide (8–32 mg/kg for 21 days) suppressed MMP-13 and MMP-3, which are responsible for degrading the extracellular matrix and are related to cartilage degeneration in collagen-induced arthritic mice (Lin et al., 2007 ▶) as well as, primary human osteoarthritis and bovine chondrocytes (125-250 nM for 24 hr) (Liacini et al., 2005 ▶). Triptolide regulates inﬂammation in other ways. Triptolide and dexamethasone, an anti-inﬂammatory and anti-allergic drug, inhibit the release of a variety of pro-inﬂammatory cytokines and chemokines. Compared with dexamethasone, triptolide (1–30 nM for 24 hr) showed stronger inhibition of granulocyte colony-stimulating factor (G-CSF), but it did not regulate the release of IL-6 or alter MIP-1β expression levels (Lu et al., 2006 ▶).

In inflammatory bowel disease C3H.IL-10 -/- mice, triptolide (0.0035 mg/ml for 8 weeks) inhibited IL-6/STAT3 pathways, which are involved in autoimmune diseases, and the expression of IL-17 to ameliorate inﬂammatory bowel diseases (Li et al., 2010 ▶). Additionally, in intervertebral disc degeneration, triptolide (50 nM for 24 hr) strongly suppressed IL-6/8, matrix metalloproteinases (MMPs), prostaglandin E2 (PGE2) and toll-like receptor protein 2 /4 (TLR2/4) and altered MAPK, p38 and ERK pathways, without affecting NF-κB in human intervertebral disc cells (Klawitter et al., 2012 ▶). MAPK signaling pathways play a vital role in the regulation of inﬂammation. Surprisingly, TGF-beta activated kinase 1 (TAB1), a potential target of MAPK, and interacts with triptolide (15–30 mM for 4–18 hr) instead of TAB1- TGF-beta activated kinase 1 (TAK1) complex, inhibiting MAPK pathway activity (Lu et al., 2014 ▶). activity (Lu et al., 2014 ▶) triptolide and immunoregulation. 

The immunosuppressive activity of triptolide is well described. Triptolide acts as an immunosuppressor to prevent immune rejection after organ transplantation, even in islet allografts (Xin et al., 2010 ▶).

**Table 1:** Triptolide targets involved in anti-inflammatory effects

**Pharmacological ** **Effects**	**Target genes**	**Action**	**Model**	**Reference**
**In** **ﬂ** **ammatory reaction**	NF-κB	Inﬂammatory cytokines, NO↓IκBα↑TLR2/TLR4↑COX-2,PGs↓	Caco-2cells,RPMI-8226cells,IL-10-deﬁcient mice	((Matta et al., 2009; Hoyle et al., 2010; Yu et al., 2011; Zeng et al., 2011; Geng et al., 2012)
	COX-2 miR155	TNFα↓ Inﬂammatory cytokines↓	A549cells IL-10deﬁcient mice	(Wu et al., 2013a)
	MMPs	IL-1α,IL-1β,TNFα,IL-6↓	Mouse macrophages, arthritis mice	(Lin, Sato, and Ito, 2001; Lin et al., 2007)
	Smad2	InhibitionofSmad2	NRK-49Fcells	(Zhu et al., 2010)
	IL-6/STAT3 Jak/STAT	IL-17,IFN-γ↓	IL-10-deﬁcient Mice, keratinocytes	(Li et al., 2010; Hongqin et al., 2011)
				
	MAPK,ERK	IL-6/8,PGE2,TLR2/4↓	Human intervertebral disc cells	(Klawitter et al., 2012)
	TAB1	Inhibition of inﬂammation	Macrophages	(Lu et al., 2014)
**Immunoregulation**	STAT3	IL-17↓	Th17cells	(Krakauer et al., 2005)
	NF-κB,PI3K-AKT	CCR7,CCL19/MIP-3β↓IL-10↑	Dendritic cells	(Liu et al., 2007; Yan et al., 2012)
	C3,CD40,B7h	TNFα↓	Human proximal tubular epithelial cells	(Hong et al., 2002)

Nitric oxide (NO); Cyclooxygenase (COX); Prostaglandins (PGs); Mothers against decapentaplegic 2 (SMAD2); Mitogen-activated protein kinase (MAPK), Extracellular-signal-regulated kinase (ERK), Phosphoinositide 3-kinase (PI3K), Chemokine (C-C Motif) Receptor 7 (CCR7), Chemokine (C-C motif) ligand 19 (CCL19), Macrophage Inflammatory Protein 3 beta (MIP-3β).

***Triptolide and lysosomal-mediated programmed cell death***

MCF-7 cells are human breast adenocarcinoma cells. They are good model for studying apoptosis-resistant breast cancer because they lack caspase-3 protein, a key executioner protein in the intrinsic and extrinsic apoptotic pathways (Janicke et al., 1998 ▶). We demonstrated that MCF-7 cells treated with triptolide undergo an atypical apoptotic death that is dependent on lysosomal membrane permeabilization (LMP) because they lack caspase-3 (Owa et al., 2013 ▶). 

This cell death was accompanied by chromatin condensation, overexpression of cleaved caspase-7 and caspase-9 proteins, and upregulation of cathepsin B in the cytosolic fractions of experimental cells (Owa et al., 2013 ▶). To our knowledge, this was the first report in the literature on triptolide-induced lysosomal membrane permeability as an anticancer treatment. We have detected cathepsin B in the cytosol of triptolide-treated MCF-7 cells via immunofluorescence staining (Owa and Halaby, unpublished results). Our results suggest that triptolide may also induce its anti-inflammatory effects and its effects on autoimmune diseases via an autophagic mechanism. Support for this notion comes from a recent study conducted by Krosch and colleagues (Krosch et al., 2013 ▶). They evaluated LC3 protein expression, a hallmark protein of autophagy. These finding along with those from our lab suggest that triptolide induces cell death via a lysosomal-regulated pathway. Furthermore, a recent study showed that triptolide can sensitize cancer cells to tumor necrosis factor related apoptosis-inducing ligand (TRAIL)-mediated cell death via two pathways (Chen et al., 2014 ▶). 

Chen et al. ( 2014) ▶ demonstrated that triptolide downregulates the pro-survival FLICE-like inhibitory protein (cFLIP), upregulates death receptor 5 (DR5), and activates LMP and mitochondrial membrane permeabilization. 

**Efficacy of triptolide**

**Efficacy of triptolide in clinical trials**

Thunder god vine *Tripterygium wilfordii* Hook F (TWHF) is a perennial plant (Celastraceae) indigenous to Southern China. TWHF has been historically used in traditional Chinese medicine to treat inflammatory and autoimmune diseases such as rheumatoid arthritis, psoriatic arthritis, systemic lupus erythematosus, and Behcet's disease (Lipsky and Tao, 1997 ▶). Triptolide, the main diterpenoidtriepoxide isolated from TWHF, has profound anti-inflammatory, immunosuppressive, and anti-proliferative activities (Qiu and Kao, 2003 ▶). When administered orally at 0.6–2.5% of LD50 (1.278 mg/kg), triptolide suppressed inflammation and cartilage destruction in collagen-induced arthritis mice (Lin et al., 2007 ▶). 

During the past three decades, more than a thousand patients with different autoimmune and inflammatory diseases have been treated with extracts of TWHF in China in clinical trials and practice. It has been reported that ankylosing spondylitis (Ji et al., 2010 ▶), psoriatic arthritis (Xu et al., 1985 ▶), and chronic nephritic syndrome (Jiang, 1994 ▶) were successfully treated. In most of these studies, the T2 extract, also known as multiglycoside or polyglucoside, was frequently used in combination with other treatments. In rheumatoid arthritis (RA) patients, the multiglycoside preparation combined with low doses of methotrexate, a standard RA drug, led to a better symptom reduction with fewer side effects than higher doses of methotrexate alone (Wu et al., 2001 ▶). The mutliglycoside preparation was as effective as prednisone in the treatment of Graves’ ophthalmopathy (Wang et al., 2004b ▶) and gave substantial improvement in the symptoms of refractory pyodermagangrenosum (Li, 2000 ▶). Multiglycoside also lowered IL-6 levels and improved symptoms in Guillain-Barre syndrome patients more effectively than adrenal corticosteroid (Zhang et al., 2000 ▶). A few studies on other Tripterygium extracts have been undertaken. Treatment with Tripterygium also decreased the size of uterine leiomyomas (uterine fibroids) (Gao and Chen, 2000 ▶). 

Two studies compared different Tripterygium preparations. The multiglycoside preparation was more effective than a *T. hypoglaucum* root preparation in treating grade 1 erosive oral lichen planus, but there was no significant difference between treatments in grade 2 patients (Lin and Qi, 2005 ▶). A preparation from *T. wilfordii* leaves was just as effective as a root preparation in alleviating the symptoms of RA, with no significant difference in the occurrence of side effects (Du et al., 1998 ▶). Pure triptolide (T1) has also been tested in clinical trials. It produced improvement in 75% of psoriasis vulgaris patients in an uncontrolled study (Wu and Guo, 2005 ▶). It also decreased the levels of urinary monocyte chemo-attractant protein-1, a marker of kidney inflammation, in patients with diabetic nephropathy (Song et al., 2005 ▶) and has shown efficacy in treating nephrotic syndrome and suppressing rejection of kidney transplants (Peng et al., 2005 ▶).

There have been numerous human clinical trials using extracts, and one study that also included triptolide (**1**) for treatment of rheumatoid arthritis and other autoimmune conditions (Tao and Lipsky, 2000 ▶; Tao et al., 2001 ▶; Tao et al., 2002 ▶). In the second study, a Tripterygium extract prolonged the survival of islet grafts in patients with diabetes (Zhang et al., 1994 ▶). Pure triptolide (**1**) has also shown significant activity in animal models like adjuvant-induced arthritis model and allograft models (Qiu and Kao, 2003 ▶; Zhu et al., 2004 ▶). Compounds related to **1 **are currently being evaluated for use in organ transplantation (First and Fitzsimmons, 2004 ▶). The effects of triptolide on Crohn’s disease was investigated in a small clinical trial with 20 patients (Ren et al., 2007 ▶). This study demonstrated significant decreases in serum levels of C-reactive protein, TNF-α, and IL-1β (Ren et al., 2007 ▶). A recent report demonstrated that triptolide ameliorated the pathological inflammation associated with Crohn’s Disease (Li et al., 2014 ▶). Another clinical trial showed that triptolide was a promising immunosuppressive agent for the treatment of IgA nephropathy (Chen et al., 2010 ▶). Likewise, triptolide was found to be effective in short-term treatment of moderately severe Henoch-Schönleinpurpura nephritis (Wu et al., 2013b ▶). One clinical study showed that triptolide was effective in treating psoriasis vulgaris (Wu and Guo, 2005 ▶). Triptolide has been reported to decrease inflammation in patients with diabetic nephropathy, thereby improving renal function (Song et al., 2005 ▶). Triptolide was proven to be an effective form of treatment for asthma through inhibition of IL-5 production (Wang and Zhang, 2001 ▶) More clinical studies, with larger sample sizes, are warranted to examine the efficacy of triptolide and its analogues as treatment options for inflammatory and autoimmune conditions.

**Efficacy of triptolide in multiple sclerosis**

Experimental autoimmune encephalomyelitis (EAE) is a demyelinating disease of the CNS that serves as an animal model for human multiple sclerosis (MS) (Martin et al., 1992 ▶). It is typically induced in rodents by immunization with various myelin-derived antigens, for example, proteolipid protein (PLP) or CNS homogenate (Whitham et al., 1991 ▶). The signs of EAE in mice are varied and resemble different clinical manifestations seen in humans (Vanderlugt and Miller, 2002 ▶). Cytokines play an important role in the pathogenesis of MS as evidenced by altered cytokine profiles in the CNS (Brosnan et al., 1995 ▶). Recent discovery described Th17 cells as a distinct subtype from Th1 and Th2 cells that mediate inflammatory pathology in EAE downstream of IL-1 (Sutton et al., 2006 ▶). Understanding the mechanisms of cytokine-mediated damage is necessary to design therapies that promote oligodendrocyte and axon survival and prevent irreversible chronic disability in both EAE and MS.

 It has been demonstrated that the therapeutic effects of triptolide in EAE are mediated via induction of a major stress protein, heat shock protein 70 (HSP70) and stabilization of NF-κB /IκBα transcriptional complex (van Eden, 2009 ▶; Kizelsztein et al., 2009 ▶). Another study showed that LLDT-8 prevents EAE by suppressing T cell proliferation and activation (Fu et al., 2006 ▶). Likewise, Wang et al. (2008) ▶ reported that triptolide reduced inflammation and dymelination in the CNS in EAE.

**Efficacy of triptolide in diabetes**

Diabetic nephropathy (DN) is one of the common complications in diabetics and major problems facing human health. Increased urine albumin excretion is not only an indication of diabetic renal injury but an important factor in the progression of DN (Jefferson et al., 2008 ▶). Evidence has confirmed that triptolide could inhibit inflammatory reaction to reduce the level of urinary monocyte chemo-attractant protein-1 (MCP-1) and thus improve renal function (Song et al., 2005 ▶). Triptolide was shown to prevent autoimmune diabetes in non-obese diabetic (NOD) mice (Huang et al., 2013 ▶). Additionally, triptolide prolonged islets allografts in diabetic C57BL/6 mice or spontaneously diabetic NOD mice (Xin et al., 2010 ▶). 

**Efficacy of triptolide in lupus nephritis**

A recent study was conducted to evaluate the therapeutic effects of triptolide on established nephritis in the (NZB X NZW) F_1_ mouse (Tao et al., 2008 ▶). Tao et al. found that triptolide remarkably improved renal disease , and there was greater survival of (NZB X NZW) F_1_ mice treated with triptolide than mice treated with vehicle (Tao et al., 2008 ▶). These results support the conclusions that the therapeutic value of extracts of TWHF in lupus nephritis can be due to triptolide, that cooperation between components is not required for therapeutic benefit, and that either diterpenoid could be an effective therapy for lupus nephritis. The mechanism by which triptolide and tripdiolide exerted their therapeutic benefit in (NZB X NZW) F_1 _mice with lupus nephritis could be related to their immunosuppressive and direct anti-inflammatory actions. It has been documented that triptolide inhibits the up-regulation of multiple pro-inflammatory genes, including IL-2, IFNγ, TNF, IL-6, COX-2, and iNOS (Tao et al., 1995 ▶; Tao et al., 1996 ▶; Lu et al., 2006 ▶).

**Efficacy of triptolide in Crohn’s disease**

 Currently, there are few therapeutic options available for patients with Crohn’s disease. The use of a natural product such as triptolide that may have fewer side effects than conventional drugs is attractive. Recent reports suggest that triptolide triggers apoptosis in IL-10-deficient mice with colitis and in lamina propria mononuclear cells of the gut via IL-6/ signal transducer and activator of transcription 3 (STAT3)/ suppressor of cytokine signaling 3 (SOCS3) signaling pathway (Li et al., 2010 ▶; Li et al., 2013 ▶). Triptolide is reported to ameliorate the effects of Crohn’s disease by inhibiting the Toll-like receptor/NF-κB signaling pathway (Yu et al., 2011 ▶). The therapeutic effects of triptolide in IL-10-deficient mice with colitis are presumably mediated via suppression of  TNFR2 expression and NF-κB activation (Wei et al., 2008 ▶).

**Triptolide toxicity**

 Although *Tripterygium wilfordii* possesses a variety of bioactivities and pharmacological effects both *in vivo* and *in vitro*, it has been restricted in clinical application due to repeated reports of multi-target toxicity. For example, commercial preparations of *T. wilfordii* are responsible for 633 adverse reactions, including 53 severe cases, that involved reproductive toxicity, hepatotoxicity, and renal cytotoxicity, among other outcomes (Li et al., 2014 ▶). Additionally, 271 patients with rheumatoid arthritis reported side effects of *T. wilfordii*, namely digestive tract symptoms and irregular menstruation (Zhou et al., 1999 ▶).

Triptolide-induced (10–2560 nM for 6–24 hr) suppression of superoxide dismutase and glutathione was accompanied by downregulation of NF-E2-related factor 2 (Nrf2), a natural antioxidants in the body. siRNA knockdown of Nrf2 aggravated nephrotoxicity in NRK-52E cells (Li et al., 2012 ▶). Moreover, triptolide (200–400 μg/kg for 28 days) induced mitochondrial membrane depolarization in female Sprague–Dawley rats, leading to liver damage with microvesicular steatosis and hyperlactacidaemia, was accompanied by an increase in reactive oxygen species (ROS) and the nicotinamide adenine dinucleotide reduced/oxidized ratio (NADH/NAD^+^) (Fu et al., 2011 ▶).

Additionally, treatment of proximal tubular epithelial cells with triptolide (100–400 μg/kg for 28 days) changed the paracellular permeability and localization of zonulaoccludens 1 protein (ZO-1) and induced proximal tubular damage (Sun et al., 2013 ▶). In terms of reproductive toxicity, triptolide (5–20 nM for 24 hr) inhibited the production of estradiol through down-regulating Human chorionic gonadotropin (HCG) and cyclic adenosine monophosphate (cAMP) via disruption of the cAMP/protein kinase A (PKA) pathways in rat granulosa cells (Zhang et al., 2012 ▶). Although there is no clear explanation for the wide range of target organs that can be adversely affected by this natural product, these results provide novel directions for further studies on triptolide toxicity.

**Anti-cancer effects of triptolide**

Cancer is promoted by deregulation of the cell cycle. Triptolide has been demonstrated to inhibit tumor cell proliferation. It has been shown to induce S-phase arrest by upregulating p21 and p27 while downregulating cyclin A protein expression levels (Meng et al., 2014 ▶). Triptolide activates pro-apoptotic proteins in tumor cells. Specifically, the cell death induced by triptolide is characteristic of the canonical apoptotic pathway and involves the activation of caspase-3, caspase-9, and poly ADP ribose polymerase  (PARP) (Hu et al., 2014 ▶). Triptolide’s inhibitory effect on the HSP70 gene presumably causes cancer cells to become more susceptible to stress-induced cell death (Westerheide et al., 2006 ▶). Triptolide also functions as an anti-angiogenic agent to inhibit metastasis (He et al., 2010 ▶). The anti-angiogenic properties of triptolide are mediated by downregulation of tyrosine kinase, endothelial (Tie2) and vascular endothelial growth factor receptor 2 (VEGFR-2) (He et al., 2010 ▶). A recent report showed that triptolide can induce cell death in breast cancer cells by decreasing the expression levels of β-catenin (Shao et al., 2014 ▶). In a study conducted by Liu et al. (Liu et al., 2014 ▶), triptolide was demonstrated to downregulate protein markers in pancreatic cancer stem cells and to reverse the epithelial-mesenchymal transition in these cells. *in vivo* experiments have also demonstrated triptolide’s therapeutic efficacy in several models including cholangiocarcinoma in a hamster model (Phillips et al., 2007 ▶) and xenografts of human melanoma, breast cancer, bladder cancer, gastric carcinoma (Yang et al., 2003 ▶), pancreatic cancer (Phillips et al., 2007 ▶) and neuroblastoma in nude mice (Antonoff et al., 2009 ▶). Further studies will undoubtedly shed light on the molecular pathways that are involved in triptolide’s anti-tumorigenic effects.

## Conclusions

The autoimmune and anti-inflammatory properties of triptolide make it an attractive agent to treat autoimmune disorders. The adverse effects of triptolide can be reduced by utilizing combinatorial strategies, such as the application of a protective agents or nanoparticle delivery-based systems, determination of the toxicity dosage range and establishment of a toxicity warning system. However, more studies are needed to understand the mechanisms that modulate the toxic effect of triptolide. In particular, more stringent randomized double-blind clinical trials are needed. We hope that further studies regarding the efficacy and toxicity of triptolide will clarify its function and mode of action, and that triptolide will be a source of a novel generation of effective anti-inflammatory drugs.
